# MiR-27a-3p functions as an oncogene in gastric cancer by targeting BTG2

**DOI:** 10.18632/oncotarget.10460

**Published:** 2016-07-07

**Authors:** Lin Zhou, Xin Liang, Lingling Zhang, Liyan Yang, Norio Nagao, Hongkun Wu, Chang Liu, Shengchao Lin, Guoxiang Cai, Jianwen Liu

**Affiliations:** ^1^ Department of Laboratory Medicine, Changzhen Hospital, Second Military Medical University, Shanghai 200003, China; ^2^ State Key Laboratory of Bioreactor Engineering & Shanghai Key Laboratory of New Drug Design, School of Pharmacy, East China University of Science and Technology, Shanghai, 200237, China; ^3^ Department of Life and Environmental Sciences, Prefectural University of Hiroshima, Shobara, 727-0023 Japan; ^4^ Department of Colorectal Surgery, Fudan University Shanghai Cancer Center, Shanghai 200032, China

**Keywords:** miR-27a-3p, apoptosis, BTG2, gastric cancer, cell proliferation

## Abstract

microRNA-27a (miR-27a) is frequently dysregulated in human carcinoma, including gastric cancer. The B-cell translocation gene 2 (BTG2) has been implicated in gastric carcinogenesis. However, till now, the link between miR-27a and BTG2 in gastric cancer has not been reported. Here, we found that two isoforms of mature miR-27a, miR-27a-5p and miR-27-3p, were both frequently overexpressed in gastric cancer tissues and cell lines, whereas the expression level of miR-27-3p in gastric cancer was significantly higher than that of miR-27a-5p. And overexpression of miR-27a-3p, but not miR-27a-5p, markedly promoted gastric cancer cell proliferation *in vitro* as well as tumor growth *in vivo*. Further experiments revealed that BTG2 was a direct and functional target of miR-27a-3p in gastric cancer and miR-27a-3p inhibition obviously up-regulated the expression of BTG2. In turn, overexpression of BTG2 triggered G1/S cell cycle arrest, induced subsequent apoptosis, and inhibited C-myc activation following Ras/MEK/ERK signaling pathway, which involved in the biological effects of miR-27a-3p/BTG2 axis on gastric carcinogenesis and cancer progression. Overall, these results suggested that the miR-27a-3p/BTG2 axis might represent a promising diagnostic biomarker for gastric cancer patients and could be a potential therapeutic target in the management of gastric cancer.

## INTRODUCTION

Gastric cancer (GC) is one of the most common malignant diseases and the second leading cause of cancer-related mortalities worldwide, especially in Asian countries [1, 2]. Despite advances in treatment modalities, the prognosis for gastric cancer patients has not been significantly improved, and the overall 5-year survival rate remains as poor as 10% to 40% [3]. So far, the pathogenic mechanisms contributing to gastric carcinogenesis and cancer progression are incompletely elucidated. Understanding of this process may identify potential markers and therapeutic targets for gastric cancer.

Recent years, it has been demonstrated that microRNAs (miRNAs) are involved in the development of malignant disease, including gastric cancer [4, 5]. MicroRNAs are a class of diverse, small, non-coding RNAs of 21–25 nucleotides in length that result in translational repression or degradation through targeting complementary sequences of mRNAs in the 3′-untranslated region (3′-UTR) and regulate a wide range of physiological and pathological processes including cell proliferation, differentiation, motility, apoptosis, angiogenesis and metastasis. Moreover, accumulated evidence indicates that miRNAs are aberrantly expressed in human cancers and may function as tumor suppressors or oncogenes [6].

microRNA-27a (miR-27a) is located at chromosome 19 and has been found to be frequently aberrant expressed and play functional roles in multiple tumor types including pancreatic cancer [7], breast cancer [8, 9], ovarian cancer [10], esophageal cancer [11], renal cell carcinoma [12], hepatocellular carcinoma [13], and glioma [14]. Previous studies have also shown that miR-27a is upregulated in GC tissues and may promote malignant behaviors by targeting the tumor suppressor gene, prohibitin [4, 15–17]. However, the expression profiles of two isoforms of mature miR-27a, miR-27a-5p and miR-27-3p, in gastric cancer and the biological effects of these two isoforms on gastric carcinogenesis has previously not been investigated.

The B-cell translocation gene 2 (BTG2) is a member of the anti-proliferative (APRO) gene family, which plays an important role in cell differentiation, inhibiting cell proliferation, promoting DNA repair, and inducing apoptosis in cellular carcinogenic processes [18]. As a tumor suppressor, BTG2 has been found to be down-regulated or absent in a variety of tumors, such as gastric cancer, breast cancer, and melanoma [19–21]. Moreover, dysregulation of BTG2 has been shown to contribute to gastric invasion, and metastasis [19]. Recent studies have demonstrated that BTG2 can be regulated by non-coding RNAs, including miRNAs [22–24], however its association with mature miR-27a in gastric cancer remains unknown.

In the present study, we examined the expression patterns of two isoforms of mature miR-27a, miR-27a-5p and miR-27-3p, in gastric cancer tissues and cell lines and investigated the biological effects of the major isoform of mature miR-27a, miR-27a-3p, on gastric cancer cell proliferation *in vitro* and tumor growth *in vivo*. We further identified the direct and functional target of miR-27a-3p in gastric cancer and explored the underlying molecular mechanisms of miR-27a-3p and its target gene, BTG2, and their roles in tumorigenesis and progression of gastric cancer, which may shed light on their targeted applications in cancer therapies.

## RESULTS

### miR-27a-3p and miR-27a-5p are overexpressed in gastric cancer tissues and cell lines

**Figure 1:**
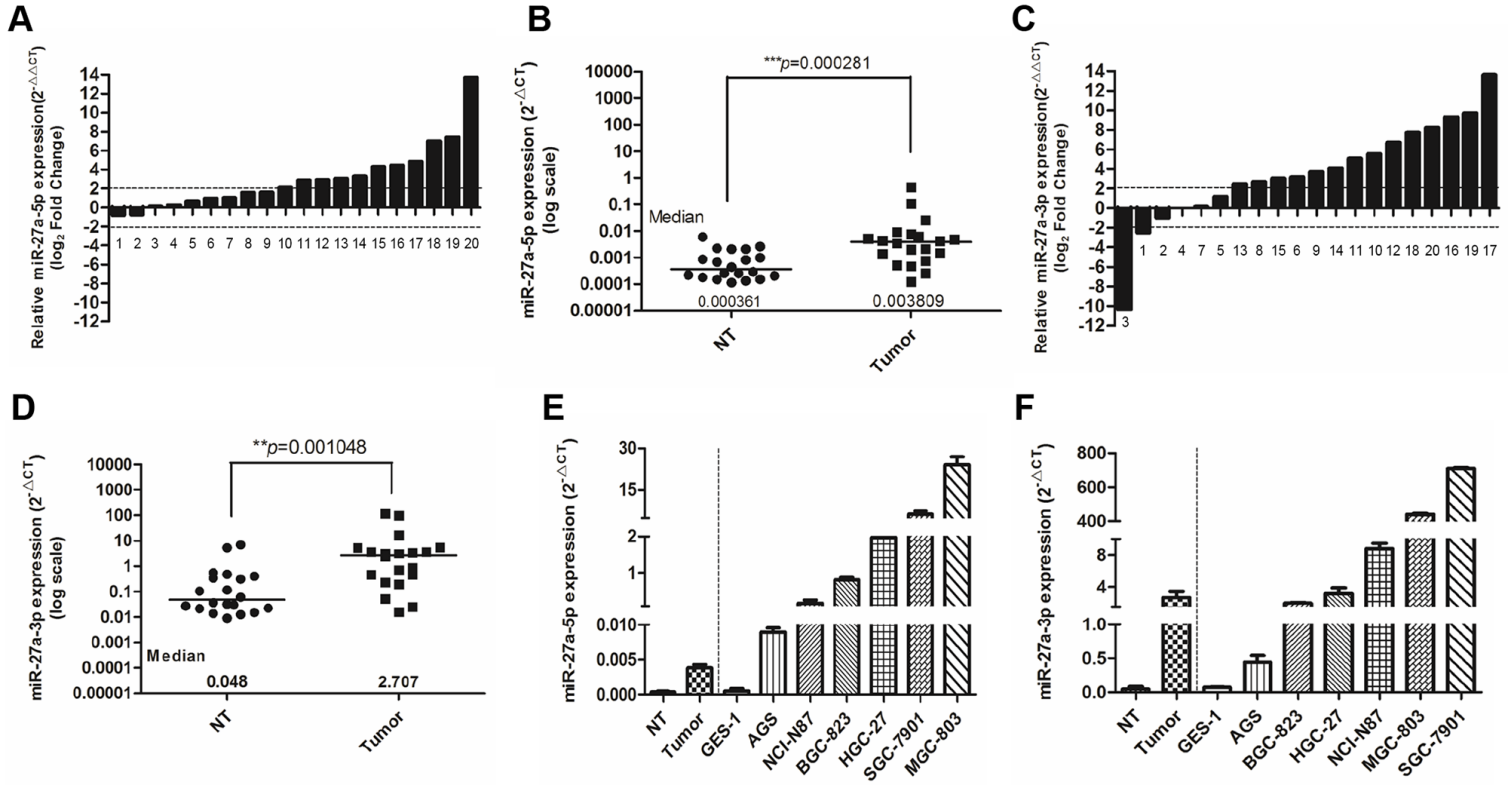
miR-27a-3p and miR-27a-5p are overexpressed in GC tissues and cell lines **A.** MiR-27a-5p expression in 20 pairs of gastric cancer tissue (Tumor) and their corresponding nontumorous tissue (NT). MiR-27a-5p expression levels were calculated by the MiR-27a-5p/U6 expression ratio (i.e., 2-ΔCt). (*p*< 0.0001, Mann–Whitney test). **B.** Comparison of MiR-27a-5p expression level between gastric cancer tissues and their corresponding non-tumorous tissues. **C.** MiR-27a-3p expression in20 pairs of gastric cancer tissue (Tumor) and their corresponding nontumorous tissue (NT). MiR-27a-3p expression levels were calculated by the MiR-27a-3p/U6 expression ratio (i.e., 2^−ΔCt^). (*p*< 0.0001, Mann–Whitney test). **D.** Comparison of MiR-27a-3p expression level between gastric cancer tissues and their corresponding non-tumorous tissues. **E.** MiR-27a-5p expression in the NT, Tumor, normal gastric cancer cell line GES-1 and gastric cancer cell lines AGS, NCI-N87, BGC-823, HGC-27, SGC-7901 and MGC-803. **F.** MiR-27a-3p expression in the NT, Tumor, normal gastric cancer cell line GES-1 and gastric cancer cell lines AGS, NCI-N87, BGC-823, HGC-27, SGC-7901 and MGC-803. Case numbers were listed below the chart of (A) and (C).

The miRbase database shows that miR-27a precursor could generate two mature miRNAs, miR-27a-5p and miR-27-3p, and miR-27-3p is the major isoform. To examine the expression patterns of these two isoforms of mature miR-27a in gastric cancer (GC), quantitative real-time PCR analysis was performed in 20 paired GC tissues and matched normal tissues. We found both miR-27-3p and miR-27a-5p had significantly increased expression in GC tissues as compared to the corresponding non-tumor samples, and the average fold-changes were greater than 2.0 (Figure 1A–1D). Notably, the fold change of miR-27a-3p median level between GC tissues and mached normal tissues (56.40, 2.707/0.048) was dramatically higher than that of miR-27a-5p (10.55, 0.003809/0.000361) (Figure 1B, 1C). In addition, consistent with the results from clinical GC samples, the levels of miR-27a-3p and miR-27a-5p were also found to be markedly up-regulated in GC cell lines (AGS, NCI-N87, BGC-823, HGC-27, SGC-7901 and MGC-803) compared with those of the normal gastric epithelial cells, GES-1, and the levels of miR-27-3p in GC cell lines were significantly higher than those of miR-27a-5p (Figure 1E and 1F). Collectively, these data indicated that two isoforms of mature miR-27a, miR-27a-5p and miR-27-3p, were both frequently overexpressed in gastric cancer, while the expression level of miR-27-3p in GC was significantly higher than that of miR-27a-5p.

### miR-27a-3p promotes gastric cancer cell proliferation and tumor growth *in vitro* and *in vivo*

**Figure 2:**
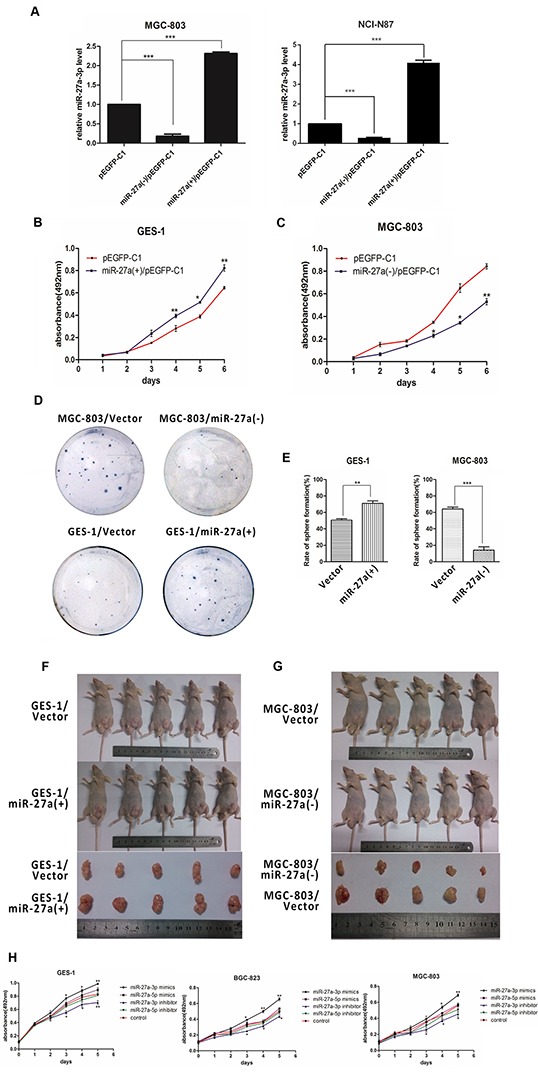
miR-27a-3p promotes GC cell growth *in vitro* and *in vivo* **A.** The verification of plasmid activation in MGC803 and NCI-N87 cells. **B.** Growth curve of GES-1 cells treat with miR-27a (+)/pEGFP-C1 or pEGFP-C1. **C.** Growth curve of MGC-803 cells treat with miR-27a (−)/pEGFP-C1 or pEGFP-C1. **D.** Sphere formation assay of stable cell lines in Soft agar. **E.** Sphere formation assay of gastric or gastric cancer cells. Equal numbers cell lines were seeded into soft agar in 6-well. After 2 weeks of culture, the number of microspheres was counted. **F.** GES-1/Vector and GES-1/miR-27a(+) subcutaneous xenograft in nude mice. **G.** MGC-803/Vector and MGC-803/miR-27a(−) subcutaneous xenograft in nude mice. **H.** Growth curve of GES-1, BGC-823, and MGC-803 cells treat with miR-27a-3p mimics or inhibitor and miR-27a-5p mimics or inhibitor.

To further clarify the role of mature miR-27a in gastric tumorigenesis, we first constructed a miR-27a expression vector, pEGFP-C1-miR-27a(+), by inserting miR-27a precursor containing some flanking sequences at both sides into the pEGFP-C1 vector, and a competitive inhibitor plasmid, pEGFP-C1-miR-27a(−). Then we performed stable transfection of these vectors into NCI-N87, MGC-803, and GES-1 cells respectively. qRT-PCR was used to verify the effectiveness of the vectors. The results showed that the expression of miR-27-3p, the major isoform of mature miR-27a, was increased obviously in pEGFP-C1-miR-27a(+) transfection cells while decreased obviously in pEGFP-C1-miR-27a(−) transfection cells (Figure 2A). Cell proliferation assays that silencing miR-27a expression dramatically reduced the growth rate of MGC-803 cells, whereas miR-27a overexpression dramatically promoted the proliferation of GES-1 cells (Figure 2B and 2C). Colony formation assays further confirmed the pro-proliferation function of mature miR-27a in GC cells (Figure 2D–2E).

Next, to evaluate the in vivo effects of mature miR-27a on gastric cancer tumor growth, four groups of nude mice were inoculated with GES-1/EGFP, MGC-803/EGFP, GES-1/miR-27a(+), or MGC-803/miR-27a(−) cells. In these four groups, tumor formation was observed from the ninth day after the implantation at an interval of 3 days, and tumor weigh was measured after the mice were sacrificed on 30th day. As shown in Figure 2F and 2G, ectopic expression of mature miR-27a promoted tumorigenesis *in vivo*: the average tumor volumes and tumor weight formed by the GES-1/miR-27a(+) cells were significantly more than those formed by the GES-1/EGFP cells, while the volumes and weight of the tumors formed by the MGC-803/miR-27a(−) were significantly less than those formed by the MGC-803/EGFP cells.

Importantly, to substantiate which isoform of mature miR-27a contributes to the oncogenic activity, the mimics or inhibitors of miR-27a-5p and miR-27a-3p were performed for the same MTT assay using the validated construcs we have measured before. Similarly, we found that the mimic of miR-27-3p, but not miR-27a-5p, markedly increased the proliferation rate of BGC-823, MGC-803 and GES-1 cells (Figure 2H). It was also shown that the inhibitor of miR-27a-3p, but not miR-27a-5p, had the similar effects to the silencing expression of the pre-miRNA. Thus, these results suggested that miR-27a-3p, the major isoform of mature miR-27a, promoted cell growth in gastric cancer.

### BTG2 is a direct functional target of miR-27a-3p in gastric cancer cells

**Figure 3:**
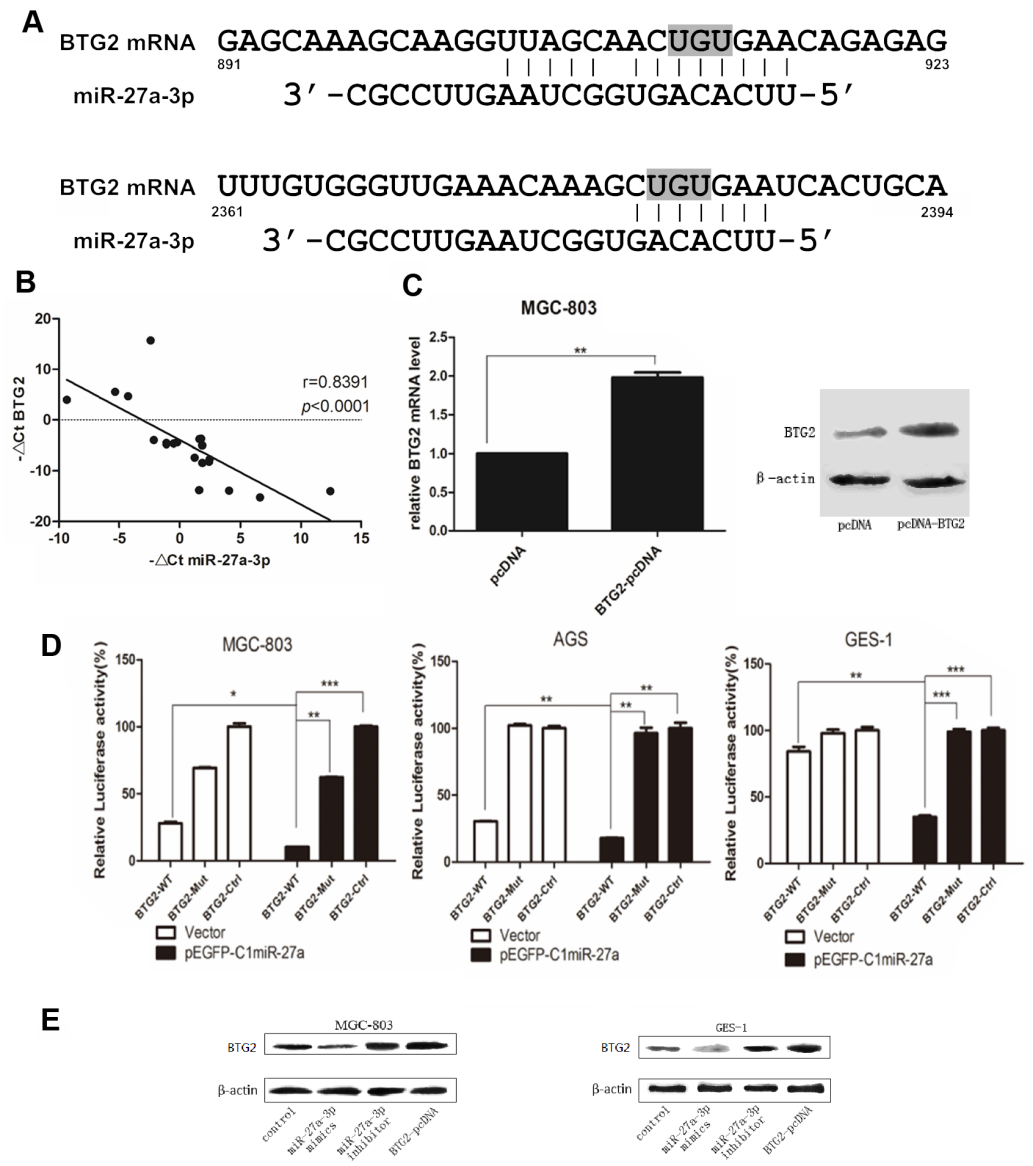
BTG2 is a direct functional target of miR-27a-3p in GC cells **A.** Schematic representation of the binding between miR-27-3p and BTG2 with mutated sites labeled with gray shading. **B.** Expression patterns of BTG2 with miR-27a-3p in gastric cancer tissues (*p*< 0.0001, spearman correlation) All data were presented as mean±SD and as representative of an average of three independent experiments. **C.** Verify the activation of BTG2-pcDNA by Real-time PCR and western blot. **D.** The effect of miR-27a-3p on BTG2 luciferase activity by dual-luciferase assay. Data were calculated by Student *t* test (^**^p< 0.01, ^***^p< 0.001). **E.** Western Blotting was performed to determine the protein expression level of BTG2 in miR-27a-3p-upregulated or miR-27a-3p-inhibited cells, β-actin was used as the loading control. All data were presented as means ± SD and as representative of an average of three measurements.

To better understand the functional mechanism of miR-27a-3p in gastric tumorigenesis, it is important to identify the direct target(s) of miR-27a-3p that might be responsible for its biological function. First, the target prediction programs miRanda, miRBase, PicTar, and TargetScan were used to predict the possible miR-27a-3p targets. Among hundreds of potential candidates, we focused on the genes which involved in cell proliferation and related to the biological functions of miR-27a-3p. BTG2 attracted our attention because its 3′-UTR contains two putative target sequences for miR-27a-3p (Figure 3A), and BTG2 is regarded as a tumor suppressor gene and closely involved in cell proliferation, apoptosis and invasion of several cancers cells, especially, of gastric cancer cells [19]. Then, to explore the relationship between BTG2 and miR-27a-3p, quantitative PCR was used to analyze the expression of miR-27a-3p and BTG2 in 20 paired clinical GC tissues. Clearly, miR-27a-3p expression inversely correlated with BTG2 mRNA expression (Figure 3B). Thus, we predicted BTG2 as a putative target gene of miR-27a-3p in GC.

Next, to identify whether BTG2 is a direct target of miR-27a-3p in GC, a dual-luciferase activity assay was performed. The wild-type BTG2 3′-UTR fragment containing two binding sites of miR-27a-3p, and the mutant of seed-region complementary sites in BTG2 3′-UTR fragment, were cloned into an luciferase reporter vector/PmirGLO vector to generate BTG2-WT and BTG2-Mut (Figure 3C). PmirGLO vector was also prepared as control vector (BTG2-ctrl). Then pEGFP-C1-miR-27a(+) plasmid was cotransfected with BTG2-WT, BTG2-Mut, or BTG2-Ctrl into AGS, MGC-803, and GES-1 cells. As shown in Figure 3D, miR-27a-3p was able to markedly inhibit the relative luciferase activity of the wild-type BTG2 3′-UTR, but did not change the activity of the mutant BTG2 3′-UTR constrcts. The result suggested that miR-27a-3p inhibited BTG2 expression via the binding site in BTG2 3′UTR. Therefore, the expression of BTG2 protein in miR-27a-3p-upregulated or miR-27a-3p-inhibited cells were investigated respectively. As a result (Figure 3E), miR-27a-3p overexpression decreased the endogenous expression of BTG2 protein, whereas miR-27a-3p inhibition increased BTG2 protein level. Taken together, theses data demonstrated that miR-27a-3p could attenuate the expression of BTG2 by directly targeting its 3′-UTR.

### miR-27a-3p/BTG2 axis regulates cell cycle progression and apoptosis in gastric cancer cells

**Figure 4:**
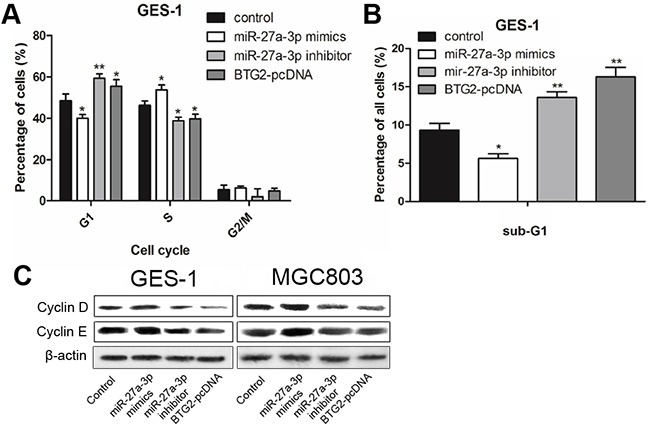
miR-27a-3p/BTG2 axis regulates cell cycle progression in GC cells **A.** Flow cytometry was performed to determine the cycle arresting of GES-1. **B.** Flow cytometry was performed to determine the sub-G1 of GES-1. **C.** Western Blotting was performed to determine the protein expression level of cyclin D and cyclin E. β-actin was used as the loading control.

It has been reported that BTG2 acts as a tumor suppressor in several human malignant tumors including gastric cancer [19]. BTG2 was found to induce cell apoptosis and trigger cell cycle arrest by inhibiting G1-S transition [20]. To determine whether the effect of miR-27a-3p on GC cell growth was mediated by BTG2, MGC-803 and GES-1 cells were transfected with miR-27a-3p mimics/inhibitor or BTG2-pcDNA for the subsequent cell-cycle or apoptosis assays. As shown in Figure 4A and 4B, overexpression of BTG2 in GES-1 cells resulted in inhibition of G1 to S phase progression. Similarly, miR-27a-3p inhibition was found to be related to G1 cell-cycle arrest, which was evidenced by the reduced percentage of S and the increased percentage of G1 and sub-G1, but miR-27a-3p overexpression could not trigger G1 cell-cycle arrest. Then we performed western blot analysis to examine the expression of cell cycle-regulatory proteins, including cyclinD1 and cyclinE1. As shown in Figure 4C, similar to the overexpression of BTG2, miR-27a-3p inhibition decreased cyclinD1 and cyclinE1 protein abundance, while miR-27a-3p overexpression increased it.

**Figure 5:**
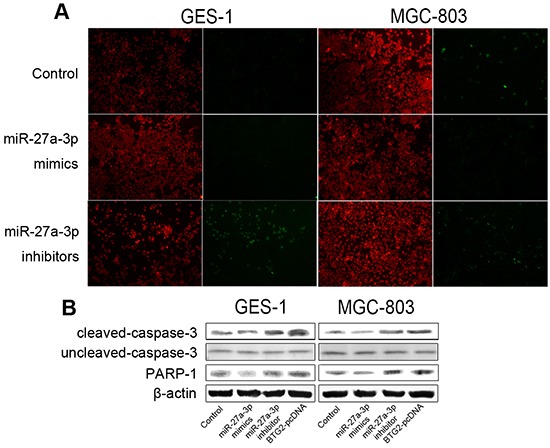
miR-27a-3p/BTG2 axis regulates apoptosis in GC cells **A.** TUNEL staining image of intervertebral disc histotomy from the first-batch SD rats. Original magnification is all 100×. **B.** Western Blotting was performed to determine the protein expression level of caspase3 and PARP1. β-actin was used as the loading control.

We further assessed the role of miR-27a-3p with BTG2 in regulating apoptosis of GC cells by TUNEL assays. As shown in Figure 5A, miR-27a-3p inhibition significantly increased the percentages of TUNEL-positive apoptotic cells when compared to miR-control, whereas miR-27a-3p overexpression slightly decreased the percentages of TUNEL-positive apoptotic cells. Next, we performed western blot analysis to examine caspase 3 cleavage and PARP1 activation in miR-27a-3p-upregulated, miR-27a-3p-inhibited or BTG2-ovexpressed cells. Consistently, the data showed that miR-27a-3p inhibition, but not miR-27a-3p overexpression, drastically caused activation of cleaved caspase 3 and PARP1 (Figure 5B). Similarly, overexpression of BTG2 was also found to induce cleaved caspase 3 and PARP1 activation. Collectively, our results indicated that miR-27a-3p/BTG2 axis modulated cell cycle profression via cell cycle-regulatory proteins and affected apoptosis by caspase 3 cleavage in GC cells.

### BTG2 inhibits C-myc activation through Ras/MEK/ERK pathways in gastric cancer cells

Since Ras/MEK/ERK signaling pathway and its downstream target, C-myc, play the key role in cell proliferation and apoptosis, and it has been reported that BTG2 is a negative regulator of Ras signaling pathway [29, 30], we sought to explore whether BTG2 could regulate C-myc expression by Ras/MEK/ERK signaling pathway.

**Figure 6:**
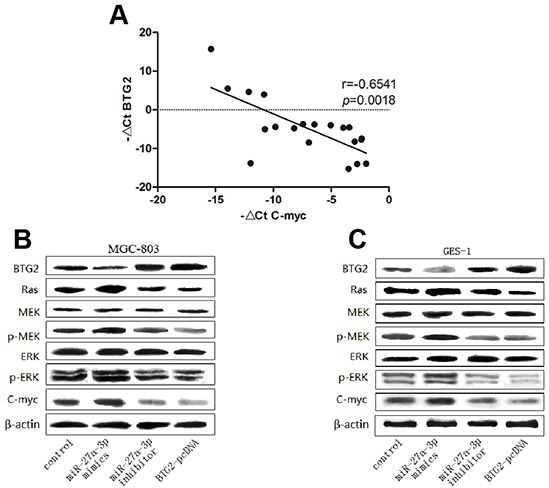
BTG2 inhibits C-myc expression through Ras/MEK/ERK pathway in GC cells **A.** Expression patterns of BTG2 with C-myc in gastric cancer tissues (*p*< 0.0001, spearman correlation) All data were presented as means±SD and as representative of an average of three independent experiments. **B.** Western Blotting was performed to determine the protein expression level of BTG2, Ras, MEK, P-MEK, ERK, P-ERK, c-Myc. β-actin was used as the loading control.

As expected, we first found a significantly inverse correlation between BTG2 expression and C-myc expression in 20 paired clinical GC samples by Real-time PCR analysis (Figure 6A). Next, to further verify the relationship between BTG2 and C-myc, we transfected MGC-803 and GES-1 cells with BTG2-pcDNA plasmids and analyzed the expression of C-myc by western blot analysis. The results showed that BTG2 overexpression could decrease the expression of C-myc (Figure 6B–6C). Moreover, to validate whether BTG2-medicated downregulation of C-myc in GC cells depends on Ras/MEK/ERK pathway, we then performed western blot analysis to examine the expression levels of several Ras/MEK/ERK downstream proteins following transfection of miR-27a-3p mimics/inhibitor or BTG2-pcDNA into MGC-803 and GES-1 cells. Also as expected, overexpression of BTG2 dramatically suppressed the expression of Ras/MEK/ERK downstream proteins (Ras, p-MEK, and p-ERK). Consistently, miR-27a-3p inhibition was found to decrease the expression of these Ras/MEK/ERK downstream molecules, whereas miR-27a-3p overexpression increased them (Figure 6B and 6C). Thus, these results demonstrated that BTG2 could inhibit C-myc expression through Ras/MEK/ERK signaling pathway in GC cells.

## DISCUSSION

Accumulated evidence has indicated that miRNAs are closely involved in the regulation of various biological and pathological processs, including tumor growth, cell invasion, and tumor metastasis [6]. Recent evidence has also shown that miRNAs are aberrantly expressed in many types of cancer and may function as tumor suppressors or oncogenes [31]. miR-27a has been found to be frequently upregulated and play functional roles in multiple tumor types including pancreatic cancer [7], breast cancer [8,9], ovarian cancer [10], esophageal cancer [11], renal cell carcinoma [12], hepatocellular carcinoma [13], and glioma [14]. Previous studies have also described aberrant expression of mature miR-27a in gastric cancer [15–17], however, very little was known about the expression profiles of its two mature isoforms, miR-27a-5p and miR-27-3p, in gastric cancer and the biological effects of these two isoforms on gastric carcinogenesis and cancer progression.

In the present study, we found that two isoforms of mature miR-27a, miR-27a-5p and miR-27-3p, were both frequently overexpressed in 20 paired clinical GC samples and GC cell lines. Herein, the expression level of miR-27-3p in GC was significantly higher than that of miR-27a-5p. Moreover, our further serial experimental results revealed that miR-27a-3p, the major isoform of mature miR-27a, promoted gastric cancer cell proliferation *in vitro* as well as tumor growth *in vivo* while its complementary strand, miR-27a-5p, did not.

As miRNAs perform their biological functions by suppressing their target genes, identifying the target genes of miR-27a-3p is important to explore the functional mechanism of miR-27a-3p in gastric tumorigenesis. Several genes have been confirmed as potential targets of mature miR-27a in a variety of cell types including Sprouty2 [7], prohibitin [4], ZBTB10 [8], FOXO1 [8], HIPK2 [10], but miR-27a has recently been reported to suppress the clonogenic growth and migration of human glioblastoma multiforme cells by targeting BTG2, a p53-inducible anti-proliferation gene and a tumor suppressor gene [32, 33]. Notably, BTG2 has been implicated in cell proliferation, apoptosis, and invasion of gastric cancer [19]. Importantly, our further analysis in gastric cancer tissues also found that miR-27a-3p expression inversely correlated with BTG2 mRNA expression. Moreover, our bioinformatics analysis revealed that BTG2 would be theoretically a potential target gene of miR-27a-3p, and BTG2 has two putative miR-27a-3p binding sites within its 3′UTR. As miRNAs usually directly inhibit the mRNA of their target genes by competitively binding with 3′UTR sites in target genes [34], we predicted that miR-27a-3p could be capable of regulating BTG2 expression via the binding site in BTG2 3′UTR. Based on a dual-luciferase reporter assay, we confirmed that miR-27a-3p directly target to the 3′-UTR region of BTG2 transcript in human gastric cancer. To further clarify it, we detected the endogenous expression of BTG2 protein after alteration of miR-27a-3p levels in GC cell lines. As expected, overexpression of miR-27a-3p reduced, but inhibition of miR-27a-3p increased, the expression levels of BTG2 protein.

Previous studies reported that overexpression of BTG2 significantly inhibited the proliferation, promoted apoptosis, and induced a G1 phase cell cycle arrest in human GC cells [19]. As the miR-27a-3p-induced cell growth we found in gastric cancer, we went on to investigate whether BTG2 functions downstream of miR-27a-3p in regulating the proliferation of GC cells. This hypothesis was confirmed by further cell-cycle and apoptosis assays, showing that miR-27a-3p inhibition, which consistent with the overexpression of BTG2, induced GC cells G1/S arrest via suppressing cyclinD1 and cyclinE1 protein abundance, and facilitated apoptosis by activating cleaved caspase 3 and PARP1. Herein, our results implied that the influence of miR-27a-3p/BTG2 axis on cell growth or proliferation might result from cell cycle arrest and subsequent apoptosis.

It has been reported that Ras/MEK/ERK signaling pathway and its downstream target, C-myc, play the key role in cell proliferation and apoptosis [29, 30, 35]. And BTG2 was found to be a negative regulator of Ras/MEK/ERK pathway [29]. Therefore, we hypothesized that miR-27a-3p/BTG2 axis could regulate C-myc expression via Ras/MEK/ERK signaling pathway in gastric cancer cells. As a result, the expression of C-myc reversely correlated with BTG2 expression in gastric cancer tissues and cell lines, and similar to miR-27a-3p inhibition, overexpression of BTG2 decreased the expression of C-myc and Ras/MEK/ERK downstream proteins (Ras, p-MEK, and p-ERK). The results from our *in vitro* experiments also suggested that the miR-27a-3p/BTG2 axis could affect C-myc activation following Ras/MEK/ERK signaling pathway in gastric cancer.

In conclusion, we found that two isoforms of mature miR-27a, miR-27a-5p and miR-27-3p, were both frequently overexpressed in gastric cancer. And overexpression of miR-27a-3p, the major isoform of mature miR-27a, promoted gastric cancer cell proliferation *in vitro* as well as tumor growth *in vivo.* Further experiments revealed that BTG2 was a direct and functional target of miR-27a-3p in gastric cancer. In turn, the biological effects of miR-27a-3p/BTG2 axis on gastric cancer cell proliferation and tumor growth resulted from G1/S cell cycle arrest, subsequent apoptosis, and C-myc activation following Ras/MEK/ERK signaling pathway. These data indicated that the miR-27a-3p/BTG2 axis might represent a promising diagnostic biomarker for gastric cancer patients and could be a potential therapeutic target in the management of gastric cancer.

## MATERIALS AND METHODS

### Reagents

All the antibodies used in the present study, including anti-MEK (catalog number, 4694) and anti-ERK (catalog number, 9102) with their Phospho-antibody (catalog number, 3598 and 4370 respectively), anti-Ras (catalog number, 3965), anti-cyclinD1 (catalog number, 2922), anti-cyclinE1 (catalog number, 4129), anti-cleaved caspase-3 (catalog number, 9661) and anti-cleaved pARP1 (catalog number, 5625), anti-β-actin (catalog number, 3700), were purchased from Cell Signaling Technology. Antibody against BTG2 was purchased from Santa Cruz Biotechnologies (catalog number, sc-33775). Dual Luciferase Reporter Gene Assay Kit was purchased fromBeyotime Biotechnology. TUNEL FITC Apoptosis Detection Kit and Cell Cycle Assay Kit were purchased from Vazyme. PCR Reagents and restriction endonucleases were purchased from Takara. PRL-TK vectors and pmirGLO Vector were purchased from Promega. PEGFP-C1 was purchased fromClontech Laboratories Inc and PcDNA 3.1(+) was purchased from Invitrogen. The miRNA mimics and inhibitor were purchased from Biotend Biotechnologies.

### Patient tissue specimens

Human GC samples and their corresponding non-tumor tissues were collected during gastric cancer surgical resection at Changzheng Hospital, Second Military Medical University and Fudan University Shanghai Cancer Center. Samples were stored and transported in liquid nitrogen. The use of these tissues was accredited by the Institutional Review Board of Second Military Medical University and Fudan University Shanghai Cancer Center.

### Cell culture

Human gastric cancer cell lines, including NCI-N87, MGC-803, AGS, BGC-823, HGC-27, SGC-7901, and human normal gastric cell line GES-1 were purchased from Cell Bank of Chinese Academy of Sciences (Shanghai, China). Cells were cultured in (RPMI)1640 medium supplemented with 10%FBS penicillin (100 U/mL) and streptomycin (100μg/mL) in a humidified environment with 5% CO_2_ at 37°C.

### Plasmid and stably transfected cell lines

The hsa-miR-27a expression vector pEGFP-C1-miR-27a(+) contains pri-miR-27a and parts of its flanking sequences, 993bp cloned into pEGFP-C1 vector digested with Xho I and ECOR I [25]. The primers used were 5′- CCGCTCGAGACTGGCTGCTAGGAAGGTG-3′ forward and 5′- GCGAATTCTTGCTGTAGCCTC CTTGTC -3′ reverse.

The hsa-miR-27a competitive inhibitor vector pEGFP-C1-miR-27a(−) was designed as a sponge of miR-27a with repeated binding sites complementary cloned into pEGFP-C1 vector digested with Hind III and Xba I. The sequences of miR-27a sponge were 5′-CCCAAGCTTACTGTGAAACTGTGAAACGTGAAACTGTGAAACTGTGAAACTGTGAATCTAGAGC-3′ forward and 5′-GCTCTAGATTTCACAGTTTCACAGTTTCACAGTTTCACAGTTTCACAGTAAGCTTGGG-3′ reverse.

GES-1/MGC-803 cells were stably transfected with pEGFP-C1-miR-27a(+) and pEGFP-C1 vector, and then cultured in the presence of 600 μg/ml G418 for 6 weeks. Clones with fluorescent label (EGFP) and G418 resistance were selected and expanded, named as GES-1/EGFP, MGC-803/EGFP, GES-1/miR-27a(+), and MGC-803/miR-27a(−), respectively. Transfection was performed using LipofectamineTM 2000 as its manufacturer's protocol.

The coding region of BTG2 cDNA was amplified by PCR using the Seamless Cloning primers containing EcoRI and XhoI restriction sites, and then cloned into the vector pcDNA 3.1(−), named pcDNA-BTG2. The primers used were 5′- AACGGGCCCTCTAGACTCGAGCAGGGTAACGCTGTCTTGTG-3′ forward and 5′- TAGTCCAGTGTGGTGGAATTCAGTTCCCCAGGTTGAGGTAT-3′ reverse. Mutated seed-region complementary sites of BTG2 3′UTR fragment were generated as follows. The primers used in the study were pair 1 (mut1), GGTTAGCAACCACGAACAGAGAGG forward and CCTCTCTGTTCGTGGTTGCTAACC reverse; pair 2(mut2), CAAAGCGTGGAATCACTGCAGG forward and CCTGCAGTGATTCCACGCTTTG reverse.

### RNA extraction and quantitative RT-PCR

Total RNA from tissues or cells were extracted by Trizol (Invitrogen Corporation) following the instructions of manufacturer. MiRNAs or mRNAs were obtained from reverse transcription-PCR using One Step PrimeScript® miRNA cDNA Synthesis Kit (Takara Bio Inc, Dalian) and TransScript® One-Step gDNA Removal and cDNA Synthesis SuperMix (TransGenBiotech, Beijing) respectively. SYBR® Premix Ex Taq™ II were employed for quantitative RT-PCR of miRNA and mRNA. U6 RNA and GAPDH were used as miRNA and mRNA internal control respectively. The primers of miR-27a-3p and miR-27a-5p used for real-time PCR were as follows: 5′-TTCACAGTGGCTAAGTTCCGC-3′and 5′-AGGG CTTAGCTGCTTGTGAGCA-3′.

### Proliferation assays and Soft-agar sphere formation assay

Cell proliferation assays of MGC-803, BGC-823 and GES-1 cells transfected with indicated vectors were performed 5 days after the transfection by incubating the cells with MTT (3-(4,5-dimethylthiazol-2-yl)-2,5-diphenyl tetrazolium bromide) [26].

Soft-agar sphere formation assay were performed with stable cell lines GES-1-EGFP, GES-1-miR-27a(+), MGC-803-EGFP and MGC-803-miR-27a(−), according to the previous reported protocol [27].

### Flow cytometric analysis

For cell-cycle analysis, cells were transfected with pcDNA-BTG2, the mimics or inhibitor of miR-27a-3p, and maintained for 48h. Cell Cycle Assay Kit (VazymeBiotech) were performed according to its manufacture's instruction and analyzed by flow cytometry using FACS Vantage (Becton Dickinson, USA). The percentage of cells in cycle phase was analyzed using wincycle software programs.

### TUNEL analysis

Briefly, cells were plated into 6-well culture plates with gelatin-coated coverslips. Adherent cells were transfected with mimics or inhibitor of miR-27a-3p for 48h. Cells were stained using TUNEL FITC Apoptosis Detection Kit as described in manufacturers' instructions and photographed under a fluorescence microscope (Leica, DMI3000B).

### Western blot analysis

Cells were transfected with mimics and inhibitor of miR-27a-3p and pcDNA-BTG2 for 48h, and then split with lysis buffer. Cellular proteins were separated in parallel lanes of 10% SDS-gels. Western blot analyses were performed as previously described [28]. β- Actin was used as a loading control.

### Luciferase reporter assay

Wild-type and mutated miR-27a-3p putative target on BTG2 3′-UTR were cloned into PmirGLO vector. Cells (2×10^4^) were seeded in 96-well plates and cotransfected with 100ng of PmirGLO-BTG2-WT or PmirGLO-BTG2-MUT constructs and pEGFP-C1-miR-27a. All samples were co-transfected with 12.5ng PRL-sv40 plasmid expressing renilla luciferase as a control for transfection efficiency. Luciferase activities were measured with the dual luciferase reporter assays at 48 h after the transfection. Transfections were performed with Lipofectamine 2000 reagent (Invitrogen).

### *In vivo* assay

For subcutaneous implantation, 5×10^6^ transfected cell lines MGC-803-EGFP, MGC-803-miR-27a(−), GES-1-EGFP and GES-1-miR-27a(+) were suspended in 100 ml PBS before injected subcutaneously into the left thigh of nude mice. Each group consisted five mice. Tumor volumes were measured from the 9^th^ day, once every three days. Tumor volumes were calculated (length × width^2^)/2 and presented as means ±SD mm^3^ [23].

### Statistical analysis

All experimental data were presented as means±standard deviation (S.D.). Two-tailed Student's *t* tests (T-test) were used to evaluate differences between groups. Mann–Whitney tests are used to evaluate the significance of differences between Clinical tumor samples and Clinical normal samples. All data were representative of an average of three independent experiments and significant differences were indicated as * *p*< 0.05; ** *p*< 0.01;*** *p*< 0.001.

## Abbreviations

miRNA: microRNA
GC: Gastric cancer
BTG2: B-cell translocation gene2
UTR: untranslated region
